# P-639. Development of antimicrobial resistance in patients with pneumonia due to *P.aeruginosa* treated with cefepime or piperacillin-tazobactam

**DOI:** 10.1093/ofid/ofae631.836

**Published:** 2025-01-29

**Authors:** Raneem H Pallotta, Samuel L Aitken, jason M Pogue, Walaiporn Wangchinda

**Affiliations:** University of Utah , Salt Lake City, Utah; Michigan Medicine, Ann Arbor, MI; University of Michigan, College of Pharmacy, Ann Arbor, MI; University of Michigan College of Pharmacy, Ann Arbor, Michigan

## Abstract

**Background:**

Pneumonia due to *P. aeruginosa* is associated with significant morbidity and mortality. Recurrent infections and the development of resistance are common. Cefepime (FEP) and piperacillin-tazobactam (TZP) are both first line treatments, yet no studies to date have compared the relative frequency of resistance development in patients treated with these two agents. This study sought to fill in this data gap.

**Table 1.**
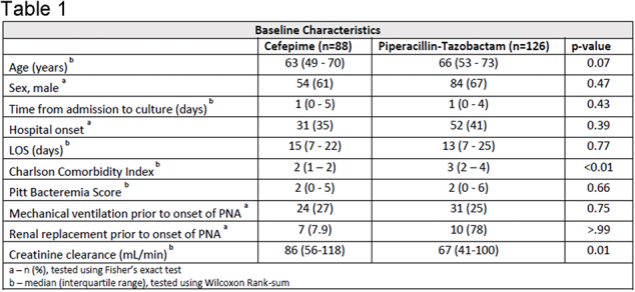
Baseline Characteristics

**Methods:**

This was a retrospective, single-center cohort study that included adult patients with culture-positive *P. aeruginosa* pneumonia treated with FEP or TZP admitted to Michigan Medicine. Pneumonia was defined as respiratory signs or symptoms and radiographic evidence of new pneumonia. Patients were eligible if they had therapy started within 24 hours of the index culture and were treated for with FEP or TZP for ≥ 3 days. Patients with unresolved infections, including COVID-19, prior to initiation of the study drug were excluded. Patients with isolates resistant to FEP and/or TZP, regardless of treatment group, were excluded. The primary outcome was development of new resistance to the study drug within 30 days, defined as a repeat culture with non-susceptibility per CLSI criteria to either FEP or TZP on repeat cultures. This was assessed using a Fine-Gray competing risk regression treating death as a competing risk and adjusting for baseline severity of illness, antimicrobial dose, and use of extended infusion.

**Results:**

214 patients (TZP, n=126; FEP, n=88) were included. Baseline characteristics were similar between the two groups, with the notable exceptions of age, creatinine clearance, and Charlson Comorbidity Index (Table 1). Frequency of need for new ICU admission (TZP 27%, FEP 31%; p = 0.65), ventilator-associated pneumonia (25% vs 28%, p = 0.75), and use of extended infusion (14% vs 17%; p = 0.70) were comparable between groups. New resistance to the study drug occurred in 13% of TZP recipients vs 6% receiving FEP (subhazard ratio [sHR] 2.61, 95% CI 0.92 – 8.97, p = 0.07).

**Conclusion:**

We identified statistically similar, but numerically increased risk of new resistance in patients with *Pseudomonas* pneumonia treated with TZP relative to FEP. These findings may help to inform definitive antimicrobial selection.

**Disclosures:**

**Samuel L. Aitken, PharmD, MPH**, Basilea: Advisor/Consultant|bioMerieux: Advisor/Consultant|Melinta: Advisor/Consultant|Shionogi: Advisor/Consultant **jason M. Pogue, PharmD**, Entasis: Advisor/Consultant|GSK: Advisor/Consultant|Melinta: Advisor/Consultant|Melinta: Grant/Research Support|Merck: Advisor/Consultant|Merck: Grant/Research Support|Shionogi: Advisor/Consultant|Shionogi: Grant/Research Support|Venatorx: Advisor/Consultant

